# First Betalain-Producing Bacteria Break the Exclusive Presence of the Pigments in the Plant Kingdom

**DOI:** 10.1128/mBio.00345-19

**Published:** 2019-03-19

**Authors:** Luis Eduardo Contreras-Llano, M. Alejandra Guerrero-Rubio, José Daniel Lozada-Ramírez, Francisco García-Carmona, Fernando Gandía-Herrero

**Affiliations:** a Department of Biochemistry and Molecular Biology A, Faculty of Biology, Regional Campus of International Excellence, Campus Mare Nostrum, University of Murcia, Murcia, Spain; b Department of Health Sciences, Universidad de las Americas Puebla, Puebla, Mexico; Max Planck Institute for Terrestrial Microbiology

**Keywords:** betalains, betalamic acid, dioxygenase, enzyme mining, pigments

## Abstract

The biosynthesis of antioxidant pigments, namely, betalains, was believed to be restricted to Caryophyllales plants. This paper changes this paradigm, and enzyme mining from bacterial hosts promoted the discovery of bacterial cultures producing betalains. The spectrum of possible sources of betalain pigments in nature is broadened by our description of the first betalain-forming bacterium, Gluconacetobacter diazotrophicus. The enzyme-specific step is the extradiol cleavage of the precursor amino acid l-dihydroxyphenylalanine (l-DOPA) to form the structural unit betalamic acid. Molecular and functional work conducted led to the characterization of a novel dioxygenase, a polypeptide of 17.8 kDa with a *K_m_* of 1.36 mM, with higher activity and affinity than those of its plant counterparts. Its superior activity allowed the first experimental characterization of the early steps in the biosynthesis of betalains by fully characterizing the presence and time evolution of 2,3- and 4,5-seco-DOPA intermediates. Furthermore, spontaneous chemical reactions are characterized and incorporated into a comprehensive enzymatic-chemical mechanism that yields the final pigments.

## Abstract (precis)

Several studies have demonstrated the health-promoting effects of betalains due to their high antioxidant capacity and their positive effect on the dose-dependent inhibition of cancer cells and their proliferation. To date, betalains were restricted to plants of the order Caryophyllales and some species of fungi, but the present study reveals the first betalain-producing bacterium, as well as the first steps in the formation of pigments. This finding demonstrates that betalain biosynthesis can be expanded to prokaryotes.

## INTRODUCTION

Betalains are pigments classified into two groups, the violet betacyanins and the yellow betaxanthins, which in addition present green fluorescence (1). The combination of both types results in the wealth of colors present in plants of the order Caryophyllales (2). Betalains are molecules with a strong antiradical capacity (3). The betalamic acid resonance system is responsible for this activity, which is modulated by the condensed molecule in each individual pigment (4). Thus, betalamic acid is not only the structural backbone of betalains but also their bioactive unit. Various studies with multiple cell lines have revealed that betalains are active against the proliferation of cancer cells (5, 6). In addition, reduction of induced tumors *in vivo* has been reported in mice when they were orally administered betalain pigments (7). In humans, betalain-rich extracts promoted an anti-inflammatory response (8). Their health-promoting effect has also been reported in the animal model Caenorhabditis elegans, where betalains reduce oxidative stress *in vivo* and increase life span (9). Thus, betalains are considered phytochemicals of nutritional value with high bioactive potential (10). The biosynthetic pathway of betalains implies the formation of betalamic acid by the enzyme 4,5-dihydroxyphenylalanine (DOPA)-extradiol-dioxygenase (4,5-DODA) and its further condensation with amino acids and amines (11). 4,5-DODA catalyzes the ring opening oxidation of the molecule l-3,4-dihydroxyphenylalanine (l-DOPA) to form the intermediate 4,5-seco-DOPA, which cyclizes spontaneously to betalamic acid (2). Analogues of betalains are present in the fungi *Amanita* (12) and *Hygrocybe* (13), where betalain-related pigments exist derived from muscaflavin, a betalamic acid isomer. No evidence that bacteria may synthesize betalains exists in the literature, but our search for novel biological systems and enzyme mining from nonnative hosts able to catalyze this reaction led to establishing bacterial cultures of microorganisms and supplementing them with l-DOPA as a precursor. This paper describes the cloning, expression, purification, and molecular and functional characterization of the betalamic acid forming DOPA-extradiol-dioxygenase from Gluconacetobacter diazotrophicus, described here to be the first bacterium producing betalains in culture. The kinetics of betalamic acid, muscaflavin, and dopaxanthin formation from l-DOPA were characterized in real time using kinetic monitoring of the reaction medium by high-performance liquid chromatography (HPLC) analysis, which for the first time allows the visualization and full characterization of 2,3- and 4,5-seco-DOPA intermediates and their formation.

## RESULTS AND DISCUSSION

### Gluconacetobacter diazotrophicus cultures produce betalamic acid.

**FIG 1:**
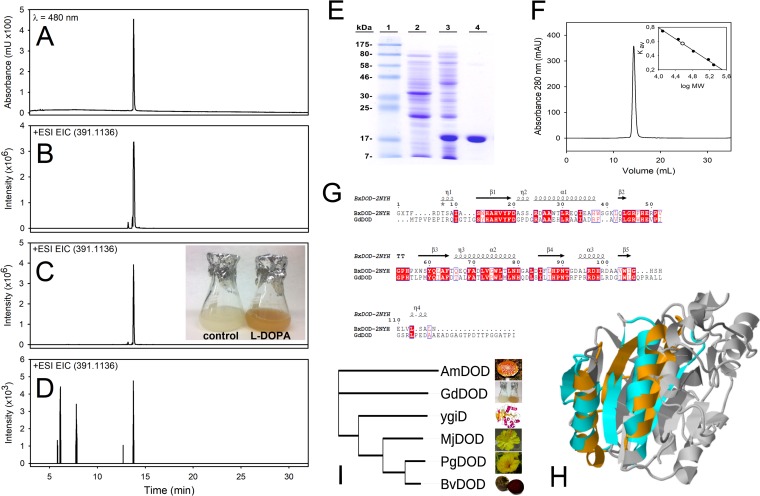
Betalain production and characterization of the novel dioxygenase from Gluconacetobacter diazotrophicus. (A to D) Detection of dopaxanthin in Gluconacetobacter diazotrophicus cultures supplemented with l-DOPA. Standard dopaxanthin was monitored by HPLC-DAD at a λ of 480 nm (A) and by HPLC-ESI-TOF MS at an extracted-ion chromatogram (EIC) of 391.1136 *m/z* (B). The same peak was detected in an EIC of *G. diazotrophicus* transformations in water (C) and culture medium (D), both supplemented with 7.6 mM l-DOPA. (E) Electrophoretic analysis for the expression and purification of recombinant GdDODA from E. coli culture. Lane 1, molecular weight markers; lane 2, soluble protein content of cells harvested prior to IPTG induction; lane 3, soluble protein content of cells harvested 20 h after IPTG induction (0.5 mM); lane 4, eluted protein after affinity chromatography purification. (F) Analysis of GdDODA by gel filtration chromatography. The profile shows a single peak corresponding to a homodimeric form. (Inset) Calibration curve and molecular mass determination. K_av_, distribution coefficient; MW, molecular weight. (G) Sequence comparison of GdDODA with the structurally characterized PDB protein 2NYH from Burkholderia xenovorans. Sequence alignment using structural information was performed with Expresso (18). Conserved blocks of amino acids are squared and strictly conserved residues are shown in red. Information was displayed with ESPript program (19). (H) Structural model for the DODA from *G. diazotrophicus* (orange and dark gray) (GdDOD) superimposed on the YgiD protein from E. coli (blue and pale gray). Orange and blue portions of the proteins indicate structural similarity according to the combinatorial extension (CE) algorithm. (I) Phylogenetic analysis of all characterized 4,5-DOPA-dioxygenases known to produce betalamic acid. Multiple-sequence alignment was performed using ClustalW2. The unrooted tree was obtained with the Phylogeny inference package from EMBL (neighbor-joining algorithm) (17, 34) using the conserved block among residues His91 and Asp122. This block contains one of the three strictly conserved histidines in the plant enzymes, which also appears in the novel dioxygenase from *G. diazotrophicus* as His101. AmDOD, GdDOD, MjDOD, PgDOD, and BvDOD, the DODAs from *Amanita muscaria*, *G. diazotrophicus*, *Mirabilis jalapa*, *Portulaca grandiflora*, and *Beta vulgaris*, respectively.

Gluconacetobacter diazotrophicus is a proteobacterium first described in roots and stems of sugarcane (14) with no evident relationship with plants of the order Caryophyllales, but its cultures supplemented with l-DOPA (7.6 mM) showed yellow coloration. HPLC analysis of yellow *G. diazotrophicus* cultures showed the presence of a peak with a retention time (R_t_) of 13.67 min and with an exact mass detected by HPLC-electrospray ionization-time of flight mass spectrometry (ESI-TOF MS) of 391.1144 *m/z*. This mass corresponded to the pigment dopaxanthin, the DOPA-derived betaxanthin first described in *Glottiphyllum longum* flowers (15), and its identity was corroborated using a real dopaxanthin standard (Fig. 1). This positive result was further investigated by harvesting *G. diazotrophicus* cells and resuspending them in water supplemented with increasing concentrations of l-DOPA until its solubility limit was reached at 7.6 mM. After 24 h, this medium showed yellow coloration, and the presence of dopaxanthin was confirmed by HPLC-ESI-TOF MS. The exact mass of 391.1144 *m/z*, corresponding to that of dopaxanthin, was detected in all the samples, and its accumulation was higher as the concentration of l-DOPA increased (see Fig. S1 in the supplemental material). The presence of l-DOPA stimulated the production of dopaxanthin, but small amounts of dopaxanthin were also detected in the absence of added l-DOPA. Therefore, Gluconacetobacter diazotrophicus produces the betalain dopaxanthin under physiological conditions in the absence of exogenous l-DOPA. Thus, G. diazotrophicus expresses a dioxygenase enzyme able to cleave the aromatic ring of l-DOPA in the same manner as the 4,5-extradiol-DOPA-dioxygenases of plant origin. This allowed us to monitor its elution by HPLC with diode array detection (DAD) and to determine the spectral maximum wavelength of the peak at a λ of 470 nm. A minor peak with the maximum λ of 405 nm and an R_t_ of 14.46 min was also detected, compatible with the presence of betalamic acid. HPLC-ESI-TOF MS and a real standard of betalamic acid were used to confirm its presence, with an exact mass of 212.0554 *m/z*.

10.1128/mBio.00345-19.2 FIG S1 Dopaxanthin accumulation in *G. diazotrophicus* cultures at different concentrations of added l-DOPA. Samples were analyzed at 24 and 48 h and showed accumulation of dopaxanthin even in the absence of exogenous l-DOPA. Download FIG S1, PDF file, 0.07 MB. Copyright © 2019 Contreras-Llano et al. 2019 Contreras-Llano et al. This content is distributed under the terms of the Creative Commons Attribution 4.0 International license.

### *G. diazotrophicus* 4,5-DODA’s sequence, expression, and purification.

A sequence with characteristics suitable to provide the dopaxanthin-forming activity described to occur in cultures of *G. diazotrophicus* was found in its genome, the WP_012222467 protein (gi 501179334), from which an optimized synthetic sequence was expressed in Escherichia coli Rosetta 2 (DE3) cells. The DODA from *G. diazotrophicus* (GdDODA) was expressed, and it accounted for 46% of the total soluble protein in the cell extract. Recombinant protein was purified by Ni^2+^-chelating affinity chromatography, subjected to SDS-PAGE, and purified to homogeneity (Fig. 1E), with a purification fold of 1.8 (Table 1), in accordance to the electrophoretic estimation of DODA expression.

**TABLE 1:** Expression and purification of *G. diazotrophicus* dioxygenase

Step	Vol (ml)	Protein (mg/ml)	Total protein (mg)	Activity^*a*^ (µM · min^−1^)	Sp act (µmol · min^−1^ · mg^−1^)	Purification fold	Yield (%)
Crude extract^*b*^	6.0	15.9	95.4	1.578	0.595	1.0	100
Ni^2+^ chromatography	7.0	4.5	31.8	0.823	1.087	1.8	61

a Activity was determined using a 50-µl protein solution under the assay conditions.

b Crude extract was obtained from a cellular paste harvested from a 0.5-liter culture.

### Molecular and structural characterization of *G. diazotrophicus* 4,5-DODA.

HPLC-ESI-TOF MS mass spectra showed a single peak with a molecular mass of 17.822 kDa, consistent with the molecular weight calculated using the protein sequence (17.8 kDa). Peptide mass fingerprint analysis further support the identity of this protein (Table 2; Fig. S2). In addition, *G. diazotrophicus* 4,5-DODA was determined to be a dimer under native conditions after gel filtration because different samples from 2.5 μg up to 0.39 mg eluted as a single peak with a molecular mass estimated at 36.5 kDa (Fig. 1F). These results further support the sequence homology found for GdDODA. We determined a 46% identity (56.6% similarity, local alignment) (Fig. 1G) to a structurally characterized enzyme from Burkholderia xenovorans Lb400 (16, 17), a dioxygenase (Protein Data Bank [PDB] accession number 2NYH) that was also demonstrated to be a dimer, which is the closest homolog structurally characterized. By structurally assisted sequence comparison, GdDODA residues were assigned to specific secondary motifs (18, 19). A three-dimensional modeling of the novel enzyme from *G. diazotrophicus* was performed by the comparative modeling engine ProMod3 (20, 21) (see Text S1 [materials and methods] in the supplemental material) and then used for comparison with the only crystallized protein known to form betalamic acid from l-DOPA in enzyme assays, the protein YgiD (PDB accession number 2PW6) from Escherichia coli, a homologue of the plant enzymes (22) (Fig. 1H). Structural comparison (23) shows how both enzymes have common local structural features. Despite the protein size differences, the small monomer of GdDODA superimposes well with one portion of the YgiD protein, and the structures composed of the amino acids Gly28-Asp41, Val45-Pro57, His58-Thr59, Leu60-Ala66, Phe67-His83, and Gln84-Pro97 in the *G. diazotrophicus* sequence are also present in the plant homologue protein.

**TABLE 2:** Main peptides identified to fully characterize the protein^*a*^

Peptide identified	*m/z*
(–)MTPVPEPIRQIGTIGSYHAHVYFDGPDGR(A)	3,210.58
(R)QIGTIGSYHAHVYFDGPDGR(A)	2,190.04
(R)DGIWLGQPRALLGSR(L)	1,638.91
(R)DGIWLGQPR(A)	1,041.55
(–)MTPVPEPIR(Q)	1,039.56
(R)AAIADR(F)	616.34
(R)DHLR(D)	540.29

a Its peptide mass fingerprint (PMF) was determined by MALDI-TOF analysis after trypsin digestion. Amino acids in parentheses correspond to the theoretical residue after trypsin digestion. (–), the beginning of the sequence.

10.1128/mBio.00345-19.1 TEXT S1 Details about structure modelling. Download Text S1, PDF file, 0.1 MB. Copyright © 2019 Contreras-Llano et al. 2019 Contreras-Llano et al. This content is distributed under the terms of the Creative Commons Attribution 4.0 International license.

10.1128/mBio.00345-19.3 FIG S2 Sequence coverage of the detected fragments identified in the peptide mass fingerprint of GdDODA. Download FIG S2, PDF file, 0.06 MB. Copyright © 2019 Contreras-Llano et al. 2019 Contreras-Llano et al. This content is distributed under the terms of the Creative Commons Attribution 4.0 International license.

### Kinetic characterization.

**FIG 2:**
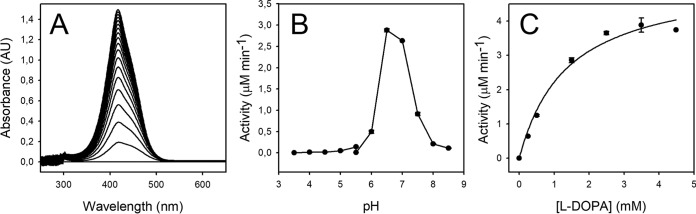
*G. diazotrophicus* dioxygenase activity characterization. (A) Spectral evolution of the transformation of DOPA (2.5 mM) by the addition of pure 4,5-DODA enzyme to the reaction medium. Spectra were recorded at 10-min intervals for 180 min, using a scanning speed of 2,000 nm · min^–1^. (B) Effect of pH on dioxygenase activity. Reactions were performed with 2.5 mM l-DOPA in 50 mM sodium acetate buffer for pH values ranging from 3.5 to 5.5 and in 50 mM sodium phosphate for pH values ranging from 5.5 to 8.5. (C) Enzyme activity dependence on l-DOPA concentration measured in 50 mM sodium phosphate buffer, pH 6.5. AU, arbitrary units.

The addition of the novel *G. diazotrophicus* enzyme to a reaction media containing l-DOPA produced a yellow coloration with a λ_max_ of 414 nm (Fig. 2A). The optimum pH for the DOPA-dioxygenase activity was determined to be pH 6.5 (Fig. 2B), and this pH was used to characterize the kinetic parameters (Fig. 2C), determined as a *K_m_* of 1.36 ± 0.31 mM and a *V*_max_ of 5.26 ± 0.43 μM · min^−1^. This *K_m_* value is lower than those obtained for *Beta vulgaris* 4.5-DODA (6.9 mM) (24), E. coli YgiD (7.9 mM) (22), and *Amanita muscaria* dioxygenase (3.9 mM) (25), making GdDODA the enzyme with the highest affinity for l-DOPA in the formation of the structural unit of betalains and the fastest one. The value for the turnover number was calculated as a *k*_cat_ of 0.50 ± 0.019 min^−1^, and the value for the specificity constant was calculated as a *k*_cat_/*K_m_* of 0.36 ± 0.01 min^−1^ · mM^−1^. Oxygen exchange was determinant in the production of betalains (Fig. S3). The production of dopaxanthin under inert atmosphere was negligible, while saturation with air provoked a production of dopaxanthin 50 times higher than that with the formation of the pigments when the exchange of oxygen was limited by diffusion. Substrates structurally related to l-DOPA, such as catechol, 4-methyl-catechol, and dihydrocaffeic acid, were also tested. These substrates showed kinetic parameters of *K_m_* 3 order of magnitude higher than the *K_m_* for l-DOPA (Table S1). In addition, theses alternative substrates showed a strong inhibition of the DOPA-dioxygenase of *G. diazotrophicus* by excess of the substrate, also called substrate inhibition (26). Inhibition curves and the kinetic mechanism are shown in Fig. S4. This makes l-DOPA the substrate most likely to be of physiological relevance. This is also supported by the presence of dopaxanthin, the final product of l-DOPA transformation in the culture medium.

10.1128/mBio.00345-19.4 FIG S3 Effect of aeration and inert atmosphere in the formation of dopaxanthin by GdDODA. Enzyme assays without stirring were considered standard conditions (not saturated air). Inert atmosphere was obtained with nitrogen gas. Download FIG S3, PDF file, 0.07 MB. Copyright © 2019 Contreras-Llano et al. 2019 Contreras-Llano et al. This content is distributed under the terms of the Creative Commons Attribution 4.0 International license.

10.1128/mBio.00345-19.5 FIG S4 Kinetic analysis of GdDODA. (A) Activity measured for the enzyme under growing concentrations of the substrates l-DOPA, dihydrocaffeic acid, 4-methyl-catechol, and catechol. l-DOPA behaves as a Michaelis-Menten substrate, while dihydrocaffeic acid, 4-methyl-catechol, and catechol present substrate inhibition kinetics. (B) Kinetic mechanism and rate equation for inhibition by excess of substrate. Download FIG S4, PDF file, 0.1 MB. Copyright © 2019 Contreras-Llano et al. 2019 Contreras-Llano et al. This content is distributed under the terms of the Creative Commons Attribution 4.0 International license.

10.1128/mBio.00345-19.9 TABLE S1 Kinetic analysis of GdDODA with different substrates. Strong inhibition by an excess of substrate was shown for dihydrocaffeic acid, 4-methyl-catechol, and catechol. Download Table S1, PDF file, 0.1 MB. Copyright © 2019 Contreras-Llano et al. 2019 Contreras-Llano et al. This content is distributed under the terms of the Creative Commons Attribution 4.0 International license.

### Mass spectrometry analysis of reaction products and intermediates.

**FIG 3:**
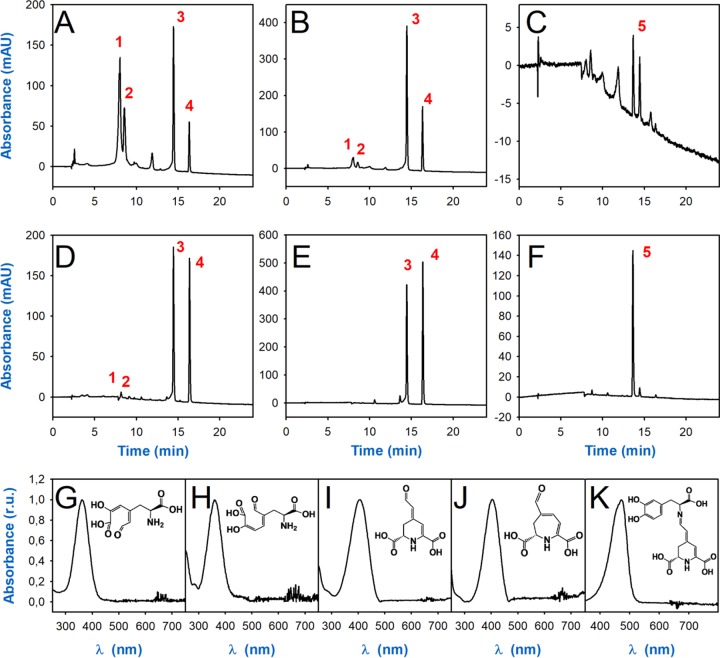
HPLC analysis of reaction products formed by *G. diazotrophicus* dioxygenase and derived compounds. (A, B, C) Chromatograms obtained at a λ of 360 nm (A), a λ of 405 nm (B), and a λ of 480 nm (C) for a reaction medium containing 2.5 mM l-DOPA and 10 mM solvent AA in phosphate buffer (50 mM, pH 6.5) at 25°C 3.3 h after the addition of the purified enzyme. (D, E, F) The same reaction monitored at a λ of 360 nm (D), a λ of 405 nm (E), and a λ of 480 nm (F) 31.5 h after the reaction was triggered. (G to K) Normalized spectra shown correspond to peak 1 (4,5-seco-DOPA) (G), peak 2 (2,3-seco-DOPA) (H), peak 3 (betalamic acid) (I), peak 4 (muscaflavin) (J), and peak 5 (dopaxanthin) (K). r.u., relative units.

The nature of the products derived from the enzymatic activity was analyzed by HPLC. The presence of betalamic acid and dopaxanthin described above for *G. diazotrophicus* cultures was confirmed (Fig. 3, peaks 3 and 5) (3). Additionally, a peak with an R_t_ of 16.32 min and a λ_max_ of 403 nm (Fig. 3, peak 4) was found in the reaction media, consistent with the product of 2,3-DOPA-extradiol-dioxygenase activity, muscaflavin. Two additional earlier peaks with a λ_max_ of 361 nm were obtained with R_t_s of 8.04 and 8.54 min (Fig. 3, peaks 1 and 2) and identified as the 4,5-seco-DOPA and 2,3-seco-DOPA precursors. All products were characterized by ESI MS and TOF MS, confirming the nature proposed above (Table 3). Fragmentation spectra for all the reported compounds with annotations are provided in Fig. S5. The accurate ESI mass spectra showed the detection of molecular protonated ions, [M+H]^+^, with exact mass values of 230.0665 *m/z*, 212.0562 *m/z*, and 391.1141 *m/z* (experimental masses). These results are consistent with the calculated masses for the molecules 4,5- and 2,3-seco-DOPAs (230.0659 *m/z*), with a difference of 2.52 ppm, betalamic acid and muscaflavin (212.0553 *m/z*), with a difference of 2.07 ppm, and dopaxanthin (391.1136 *m/z*), with a difference of 1.25 ppm. All the different values in this analysis are below the accepted accuracy threshold for elemental composition analysis, established at 5 ppm (27). For first time, these masses are experimentally determined for readily obtained seco-DOPA intermediates, and hence a complete and unambiguous picture of intermediates and final products of the evolution of l-DOPA in the presence of DOPA-dioxygenase is obtained.

**TABLE 3:** HPLC-ESI-TOF MS analysis of the reaction products formed by *G. diazotrophicus* dioxygenase activity in water supplemented with l-DOPA at 7.6 mM

Compound	Chemical formula	[M + H]^+^ (*m/z*)	Main- daughter ion (*m/z*)	Secondary- daughter ion(s) (*m/z*)	TOF exact mass (*m/z*) (exptl)	Calculated mass (*m/z*) (theoretical)	Δppm
4,5-Seco-DOPA	C_9_H_11_NO_6_	230.2	140.0	187.1, 94.1	230.0065	230.0659	2.52
2,3-Seco-DOPA	C_9_H_11_NO_6_	230.2	140.0	94.1	230.0065	230.0659	2.52
Betalamic acid	C_9_H_9_NO_5_	212.0	166.1	138.0	212.0562	212.0553	2.07
Muscaflavin	C_9_H_9_NO_5_	212.0	166.0	149.0	212.0562	212.0553	2.07
Dopaxanthin	C_18_H_18_N_2_O_8_	391.3	347.1	301.1, 255.1	391.1141	391.1136	1.25

10.1128/mBio.00345-19.6 FIG S5 ESI-MS fragment spectra of betalains and intermediate compounds identified in this work. MS2 spectra of all compounds are provided with structures and annotations. Download FIG S5, PDF file, 0.4 MB. Copyright © 2019 Contreras-Llano et al. 2019 Contreras-Llano et al. This content is distributed under the terms of the Creative Commons Attribution 4.0 International license.

### Chemical formation and comprehensive enzymatic-chemical mechanism.

**FIG 4:**
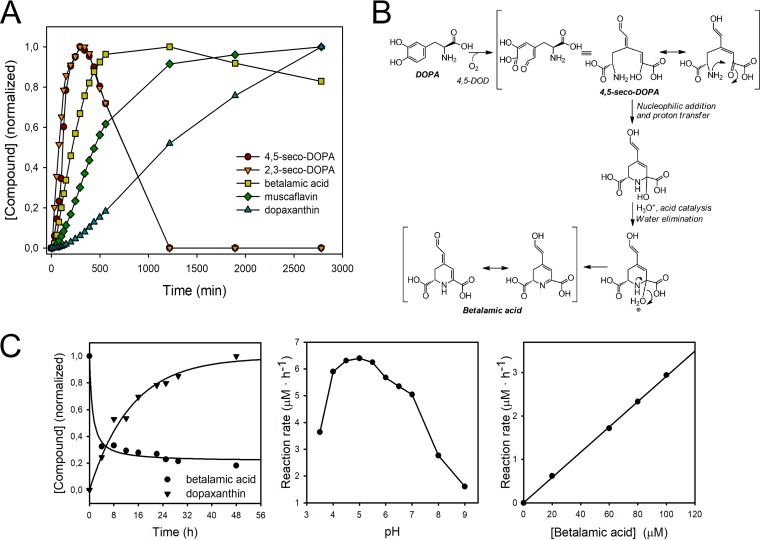
Enzymatic-chemical mechanism in the formation of betalains. (A) Time evolution of the reaction`s products formed by *G. diazotrophicus* dioxygenase activity and their derived compounds. Normalized data are based on pigment content determination by HPLC at the wavelengths 360 nm (seco-DOPAs), 405 nm (betalamic acid and muscaflavin), and 480 nm (dopaxanthin). (B) Proposed chemical reactions underlying the spontaneous cyclization of the enzyme-generated 4,5-seco-DOPA intermediate into betalamic acid. (C) Transformation of betalamic acid. (Left) Time course for the formation of dopaxanthin from betalamic acid (100 µM) and l-DOPA (3.5 mM) at pH 5.0. (Center) pH effect on the rate of dopaxanthin synthesis. (Right) Effect of betalamic acid concentration on the formation of dopaxanthin at a fixed concentration of l-DOPA (3.5 mM). Rates were calculated based on dopaxanthin concentration in the reaction medium after 12 h.

**FIG 5:**
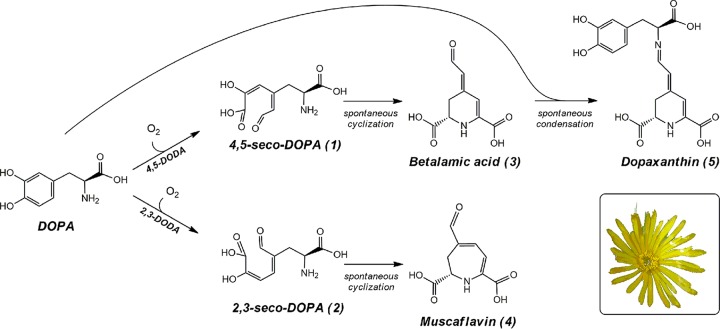
Biosynthetic scheme of betalains. The enzyme-catalyzed reactions and the chemical spontaneous cyclization and condensation ultimately yielding the flower pigment dopaxanthin are shown. The flower shown corresponds to *Glottiphyllum oligocarpum*, which produces dopaxanthin as the only pigment responsible for its coloration. Numbers in brackets correspond to the intermediates and final products described in Fig. 3.

The presence of the intermediates 4,5 and 2,3-seco-DOPAs reaches a maximum 5 h after the reaction started, and then these intermediates diminish until they disappear (Fig. 4A). Betalamic acid reaches its maximum value at 20 h and experiences a further decrease, while muscaflavin accumulates during the time in which the reaction was monitored. The formation of both betalamic acid and muscaflavin is preceded by a lag period, justified by the need for the formation of the corresponding seco-intermediates, before these molecules can be obtained. The lag period was 33 min for betalamic acid, and after its maximum, the concentration decrease was due to its condensation with intact l-DOPA molecules, resulting in the formation of dopaxanthin (Fig. 5), after a lag period of 3.35 h (Fig. 4A). Absolute concentrations of dopaxanthin, betalamic acid, and muscaflavin in the medium are provided in Fig. S6. The analysis of this chemical reaction yields a kinetic constant of 189 h^−1^ · M^−1^. The timescale obtained (Fig. 4C) is in accordance with the results shown in Fig. 4A and justifies the time needed to obtain color in developing flowers even when the initial enzyme-catalyzed reaction is finished. This chemical reaction implies a not previously considered additional time in the formation of the final pigments in betalain biosynthesis. It is a spontaneous chemical, but not immediate, reaction which depends on pH (Fig. 4C).

10.1128/mBio.00345-19.7 FIG S6 Evolution of betalamic acid, muscaflavin, and dopaxanthin in an enzymatic assay with GdDODA. Absolute concentrations are expressed in micromolar units, and conditions are as those described in the legend of Fig. 4. Download FIG S6, PDF file, 0.1 MB. Copyright © 2019 Contreras-Llano et al. 2019 Contreras-Llano et al. This content is distributed under the terms of the Creative Commons Attribution 4.0 International license.

The identification of the final product and intermediates makes GdDODA the smallest dioxygenase able to produce betalamic acid described in the literature (22, 24, 28–30). All betalamic acid-forming dioxygenases described up to now can be analyzed in terms of sequence homology and phylogeny (Fig. 1I). There is a compact clade formed by plant sequences (2) and the YgiD protein separated from *A. muscaria* dioxygenase. The dioxygenase from *G. diazotrophicus* stands apart from these two branches. An extended phylogenetic analysis was performed, and the results shown in Fig. S7 reveal the homology of *G. diazotrophicus* DODA with the sequences from *Bradyrhizobium*, *Komagataeibacter*, *Mesorhizobium*, and *Inquilinus.* All these sequences stand apart from those of previously characterized enzymes. Thus, sequence analysis reveals that GdDODA corresponds to a hitherto-unknown group of enzymes involved in this biosynthetic pathway.

10.1128/mBio.00345-19.8 FIG S7 Extended phylogenetic analysis of the novel betalain-forming dioxygenase from *G. diazotrophicus*. (A) The novel enzyme was searched against similar enzymes, and the 100 most similar sequences were used to construct a neighbor-joining phylogenetic tree (S. F. Altschul, J. C. Wootton, E. M. Gertz, R. Agarwala, et al., FEBS J 272:5101–5109, 2005, https://doi.org/10.1111/j.1742-4658.2005.04945.x; W. Li, A. Cowley, M. Uludag, T. Gur, et al., Nucleic Acids Res 43:W580–W584, 2015, https://doi.org/10.1093/nar/gkv279). The tree shows the presence of three main clades comprising species of *Bradyrhizobium* (red), *Komagataeibacter* (orange), and *Mesorhizobium* (yellow). Sequences corresponding to *Inquilinus* are shown in blue, and those corresponding to *Acetobacter* are shown in pink. (B) The block among residues His91 and Asp122 in GdDODA (see the main text for details) was used to construct a larger tree with all the characterized betalain-forming enzymes and including bacterial members of the different classes identified in panel A. In this tree, experimentally validated enzymes are labeled. The presence of betalamic acid derivatives (betalains) is also indicated. Additional sequences corresponding to bacterial members are WP_027577986 (*Bradyrhizobium*), WP_003618779 (*Komagataeibacter*), RWL99607 (*Mesorhizobium*), and WP_034835604 (*Inquilinus*). Download FIG S7, PDF file, 0.5 MB. Copyright © 2019 Contreras-Llano et al. 2019 Contreras-Llano et al. This content is distributed under the terms of the Creative Commons Attribution 4.0 International license.

Taking into account the evolution described and the nature of the intermediates characterized, the scheme shown in Fig. 4B is proposed for the “spontaneous cyclization” of the first compound in the biosynthesis of betalains. It shows how a nucleophilic addition with a concomitant proton transfer is possible and favored by the presence of the carboxylic group generated in the enzymatic cleavage of the ring. Water elimination by acid catalysis gives the structural unit of betalains. The same scheme can be proposed for muscaflavin, if one considers the different isomer forms derived from the 2,3-extradiol cleavage.

In summary, an enzymatic-chemical mechanism is described for dioxygenase formation of betalamic acid and betalains which puts together the enzyme-catalyzed reactions and the evolution of the intermediates. The one-pot experiments described show how a single enzyme can produce the final betalain product in hours. The intermediates have been followed and structurally characterized thanks to an extraordinarily high activity detected in the enzyme from *G. diazotrophicus*, the first bacterium described able to synthesize betalains. The reactions, intermediates, products, constants, and lag periods described constitute the clearest experimental evidence of the first reactions involved in the route of betalain biosynthesis, expanded now to prokaryotes.

## MATERIALS AND METHODS

### Chemicals, bacterial strains, plasmids, and enzymes.

Distilled water was purified using a Milli-Q system (Millipore, Bedford, MA, USA). HPLC-grade acetonitrile was obtained from Fisher Scientific UK (Leicestershire, United Kingdom). E. coli Rosetta 2 (DE3) cells, E. coli DH5α cells, and the plasmid pET28a were obtained from Novagen (Merck KGaA, Darmstadt, Germany). A HyperLadder 1-kb DNA ladder was obtained from Bioline Reagents Ltd. (London, United Kingdom). Restriction enzymes and the protein ladder were obtained from New England BioLabs (Ipswich, MA, USA). T4 DNA ligase was from Roche Diagnostics (Basel, Switzerland). *Pfu* DNA polymerase was purchased from Thermo Fisher Scientific Inc. (Waltham, MA, USA). A QIAprep spin plasmid miniprep kit, QIAquick PCR purification kit, and QIAquick gel extraction kit were from Qiagen (Hilden, Germany). All other chemicals and reagents were obtained from Sigma (St. Louis, MO, USA).

### Gluconacetobacter diazotrophicus culture.

Gluconacetobacter diazotrophicus was acquired from the Deutsche Sammlung von Mikroorganismen und Zellkulturen (DSMZ; the German collection of microorganisms and cell cultures) under accession number DSM5601. Active growing microorganisms were inoculated in 20-ml cultures of a specific medium that contains, per liter, 25 g d-mannitol, 5 g yeast extract, and 3 g peptone. These cultures were maintained overnight at 25°C with agitation. Afterwards, half of the cultures were centrifuged for 10 min at 5,000 × *g* and resuspended in 20 ml distilled water. The other half were kept in the culture medium. Both water and medium cultures were supplemented with various concentrations of l-DOPA (from 0 to 7.6 mM) and sodium ascorbate (15 mM) and further cultured at 25°C. Appropriate control media without l-DOPA were also cultivated at 25°C for each condition. After 2 days, the media were collected and analyzed by HPLC-ESI-TOF MS in a search for compounds derived from the synthesis of betalamic acid.

### Gluconacetobacter diazotrophicus DODA sequence and cloning.

The sequence of the protein WP_012222467, a hypothetic aromatic ring-cleaving dioxygenase from Gluconacetobacter diazotrophicus PA5, has been deposited at the National Center for Biotechnology Information (NCBI, Bethesda, MD, USA) under code gi 501179334. This sequence was used as a template to synthetically obtain the 4,5-DODA sequence from *G. diazotrophicus*, enhanced for E. coli expression (GeneArt, Regensburg, Germany). PCR amplification was performed using *Pfu* DNA polymerase and the following primers, which include the restriction sequences recognized by the enzymes NdeI and XhoI: GdDODA-F (5´TATATATACATATGACACCGGTGCCGGAA) and GdDODA-R (5´ATATATATCTCGAGTTAAATCGGGGTTGC). This amplification yielded a 457-bp product, and its length coincides with that of the entire DODA synthetic gene plus the recognized sequences for the restriction enzymes. The PCR product was digested with NdeI and XhoI, purified with a QIAquick PCR purification kit, and inserted into the expression vector pET28a downstream of the T7 RNA polymerase promoter, the expression vector was previously digested with the same restriction enzymes. This produced the recombinant plasmid PET28a-GdDODA, which encodes an additional 22-amino-acid N-terminal sequence containing a 6×His tag. The plasmid was transformed into E. coli DH5α (Novagen) electrocompetent cells and plated onto LB agar plates containing kanamycin. The resulting colonies were then analyzed by PCR. The plasmid pET28a-GdDODA was obtained from positive colonies using the QIAprep spin plasmid miniprep kit. The sequence was confirmed by DNA sequencing of the plasmid and subsequently used in further experiments.

### Expression and purification.

The GdDODA protein derived from plasmid pET28a-GdDODA was expressed in E. coli Rosetta 2 (DE3) (Novagen) and grown at 37°C in LB medium containing chloramphenicol and kanamycin to an *A*_600_ of 0.8 to 1.0. Induction was performed for 20 h with different concentrations of the inductor isopropyl-1-thio-β-d-galactopyranoside (IPTG) at different temperatures. Cells were harvested by centrifugation and resuspended in sodium phosphate buffer (50 mM, pH 8.0) with 0.3 M sodium chloride. Cell lysis was performed by sonication in a Cole-Parmer 4710 series ultrasonic homogenizer (Chicago, IL, USA). For trial scale and parameter optimization, chemical lysis was performed using BugBuster protein extraction reagent (Novagen). Recombinant protein was purified by His-select nickel affinity gel (Sigma) according to the manufacturer’s instructions and then desalted using PD10 columns (General Electric Healthcare, Milwaukee, WI, USA) and eluted into Tris-HCl (20 mM, pH 8.5) buffer. Protein was quantified using the Bradford protein assay (Bio-Rad, Hercules, CA, USA) (31), and bovine serum albumin was used as the standard to obtain a calibration curve. Samples were analyzed by sodium dodecyl sulfate-polyacrylamide gel electrophoresis (SDS-PAGE) by application to 12% polyacrylamide gels and stained using a standard Coomassie blue method.

### Gel filtration.

Samples of pure recombinant protein were applied to a Superdex 200 10/300 GL column equilibrated with sodium phosphate buffer (50 mM, pH 7.5) with 150 mM NaCl. The protein was eluted with the same buffer at a flow rate of 0.5 ml min^−1^. Elutions were performed in an Äkta purifier apparatus (General Electric Healthcare) and monitored at 280 nm. Column calibration was performed with the following protein markers (Sigma): cytochrome *c* (12.4 kDa), carbonic anhydrase (29 kDa), albumin (66 kDa), alcohol dehydrogenase (150 kDa), and β-amylase (200 kDa).

### MALDI-TOF MS protein analysis.

The matrix solution for peptide analyses was α-cyano-4-hydroxycinnamic acid (20 mg ml^−1^) in acetonitrile (CAN)-water-trifluoroacetic acid (TFA) (70:30:0.1). The peptide sample was dissolved in 0.1% TFA and mixed with the matrix solution. One microliter of this mixture was applied to the atmospheric-pressure matrix-assisted laser desorption ionization (AP-MALDI) target plate and allowed to dry. Experiments were carried out with an Agilent TOF mass spectrometer (Agilent Technologies, Santa Clara, CA, USA), equipped with an AP-MALDI ion source with an N_2_ laser (337 nm). Samples were measured in reflectron mode to identify molecular formulas based on precise mass measurements in positive mode. External calibration of the spectrometer was performed with standard peptides from the ProteoMass Peptide MALDI-MS calibration kit (Sigma). Data were recorded and processed with Agilent MassHunter Workstation software. Peptide mass fingerprint determination was carried out using Agilent Spectrum Mill software. Determination of protein absolute molecular mass was carried out using an HPLC-ESI-MS TOF system. This system comprises an HPLC Agilent VL 1100 apparatus equipped with an autosampler μ-well plate and a capillary pump connected to an Agilent 6100 TOF MS, and an electrospray ionization interface was used. The column employed was a Zorbax Poroshell 300SB-C_18_ column (1 by 75 mm with 5 μm; Agilent Technologies, Santa Clara, CA, USA). The column was operated at 60°C, and the samples were injected with a flux of 0.2 ml min^−1^. The protein was eluted with a linear gradient using water-CAN-formic acid (95:4.9:0.1) as solvent A, and water-CAN-formic acid (10:89.9:0.1) as solvent B. A linear gradient from 0% to 90% of solvent B was performed for 30 min. Protein separation was monitored at 210 and 280 nm using a multiple-wavelength detector. The mass spectrometer was operated in positive mode in the range of 100 to 2,200 *m/z*, using a capillary voltage of 3.5 kV. Nebulizer gas pressure was 30 lb/in^2^, and drying gas flux was 8 liters/min at a temperature of 350°C. External spectrometer calibration was carried out using the ProteoMass peptide MALDI-MS calibration kit (Sigma-Aldrich, St. Louis, MO, USA). Two different peptides were used as controls (cytochrome *c* and carbonic anhydrase; Sigma-Aldrich). All data were recorded and processed through the Agilent MassHunter Workstation Qualitative Analysis software (Agilent Technologies), and the intact molecular weight of the protein was obtained using the deconvolution algorithm from this software.

### Trypsin digestion.

The protein sample was prepared in 100 μl of buffer NH_4_HCO_3_ (50 mM, pH 8.0) with 0.02% ProteaseMAX surfactant (Promega, Madison, WI, USA). After that, the sample was reduced with dithiothreitol (DTT) at 10 mM and 56°C for 20 min and alkylated with iodoacetamide 50 mM at room temperature in the dark for 20 min. One microgram of proteomics-grade trypsin (Promega) was added, and the sample was incubated at 37°C for 4 h. Finally, the sample was centrifuged at 15,000 × *g* for 1 min to collect the condensate, and 0.5% TFA was added to stop the digestion. Peptides were cleaned up with C_18_ ZipTips (Millipore) and evaporated using an Eppendorf vacuum concentrator, model 5301.

### HPLC analysis of metabolites.

A Shimadzu (Kyoto, Japan) LC-20AD apparatus equipped with an SPD-M20A photodiode array detector was used for analytical HPLC separations performed with a 250- by 4.6-mm Kromasil 100 C_18_ column packed with 5-μm particles (Teknokroma, Barcelona, Spain) (32). A linear gradient was performed using water with 0.05% TFA as solvent A and acetonitrile with 0.05% TFA as solvent B. The linear gradient was performed for 24 min from 0% solvent B to 35% solvent B, the flux was 1 ml min^−1^, and the column operation temperature was 30°C. Fresh samples were analyzed after enzymatic reactions. In the case of the time course for the enzyme reaction, 1.7 ml of the reaction medium was placed in an HPLC vial with the thermostatic block set at 25°C, and injections were directly performed at different times. Formation of pigments by the purified enzyme was also assayed under an inert atmosphere (nitrogen). Oxygen exchange effect was measured with and without aeration. In this case, all samples contained 700 μl phosphate buffer (0.2 M, pH 6.5), 300 μl sodium ascorbate (100 mM), 1 ml l-DOPA (7.6 mM), 500 μl purified enzyme, and 500 μl MilliQ water up to a final volume of 3 ml. After 48 h, the samples were collected and analyzed by HPLC. In all cases, the injection volume was 50 μl.

### Electrospray ionization mass analysis of metabolites.

An Agilent VL 1100 apparatus with an LC mass selective detector (MSD) Trap was used for HPLC-ESI MS analyses. Elution conditions were analogous to those described above, using the same column. The vaporizer temperature was 350°C, and the voltage was 3.5 kV. Nitrogen at a pressure of 45 lb/in^2^ was used as the sheath gas. Samples were ionized in positive mode. The ion monitoring mode was full scan in the range *m/z* 50 to 600. The electron multiplier voltage for detection was 1,350 V. A TOF–quantitative TOF (Q-TOF) Agilent 6220 MS equipped with a dual ESI-atmospheric pressure chemical ionization (APCI) interface was used for accurate mass determinations. Samples were ionized in positive mode, using a capillary voltage of 3.5 kV. Nitrogen was used as the drying gas, the gas temperature was 350°C, flux was set at 11 liter min^−1^, and the nebulizer pressure was 40 lb/in^2^. All data were processed through the MassHunter software (Agilent Technologies).

### Absorbance spectroscopy.

Enzyme activity was determined using a continuous spectrophotometric method by measuring the absorbance due to betalamic acid and muscaflavin appearance at a λ of 414 nm (24, 25). Unless otherwise stated, the reaction medium contained 50 mM sodium phosphate buffer, pH 6.5, 2.5 mM l-DOPA, and 10 mM sodium ascorbate (AA). Measurements were performed at 25°C in 96-well plates in a Synergy HT plate reader (Bio-Tek Instruments, Winooski, VT, USA). The final sample volume was 300 μl. The plate reader detector signal was calibrated with betalamic acid solutions of known concentration. The molar extinction coefficient at 424 nm, with an ε of 24,000 M^−1^ · cm^−1^, was taken for the quantification of betalamic acid (33). Additionally, a JASCO V-630 spectrophotometer (JASCO Corporation, Tokyo, Japan) was used to measure the accumulation of reaction products over time, and the measures were carried out with a wavelength scan from 250 to 700 nm every 10 min for 3 h at 25°C. Measurements were performed in triplicate, and mean values and standard deviations were plotted. Errors associated with the results provided correspond to the residual standard deviations. Kinetic data analysis was carried out by using nonlinear regression fitting with SigmaPlot Scientific Graphing for Windows, version 10.0 (Systat Software, San Jose, CA, USA).

### Chemical transformation of betalamic acid.

The condensation of betalamic acid was kinetically characterized by determining the kinetic constant of the reaction. In the kinetic scheme for a second-order reaction in which two reactants yielded a single product following a stoichiometry of 1:1, the mass balance of the system has to be considered according to the following schemes: *v* = *k*[bet] · [DOPA] and [bet]_0_ = [bet] + [dopax], where *v* is the reaction rate, [bet] is the concentration of betalamic acid, [bet]_0_ is the initial concentration of betalamic acid before the reaction is triggered, and [dopax] is the concentration of dopaxanthin formed by the chemical reaction. By keeping constant the concentration of l-DOPA, a pseudo-first-order kinetics can be obtained as follows: *v* = *k*_app_[bet] and *k*_app_ = *k*[DOPA], where *k*_app_ is the apparent constant at a fixed DOPA concentration. The substrate concentration equation $\left[\text{bet}\right]={\left[\text{bet}\right]}_{0}\cdot e^{-k_{\text{app}}\cdot t}$, where *t* is time, can be transformed into the product accumulation $\left[\text{dopax}\right]={\left[\text{bet}\right]}_{0}\cdot \left(1-e^{-k_{\text{app}}\cdot t}\right)$. By representing the accumulation of dopaxanthin, [dopax], versus the initial concentration of betalamic acid, [bet]_0_, the apparent constant can be determined from the slope of the linear representation obtained in the graph as *k*_app_ = (Ln slope – Ln 1)/*t*, and then the kinetic constant of the second-order reaction can be determined.
