# Supplementary figures and images for: First Betalain-Producing Bacteria Break the Exclusive Presence of the Pigments in the Plant Kingdom
Source: mBio. 2019 Mar 19;10(2):e00345-19. doi: 10.1128/mBio.00345-19 (PMC6426604; doi:10.1128/mBio.00345-19)

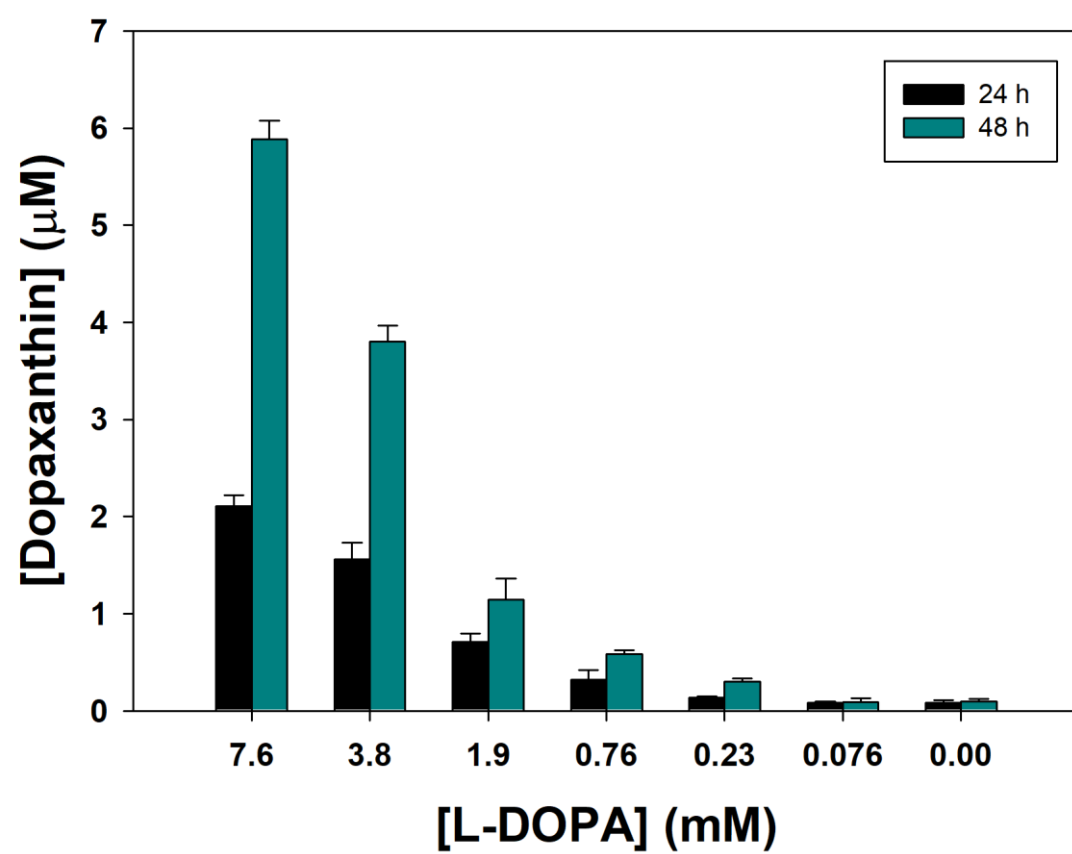

Supplement: FIG S1 [file mBio.00345-19-sf001.pdf]

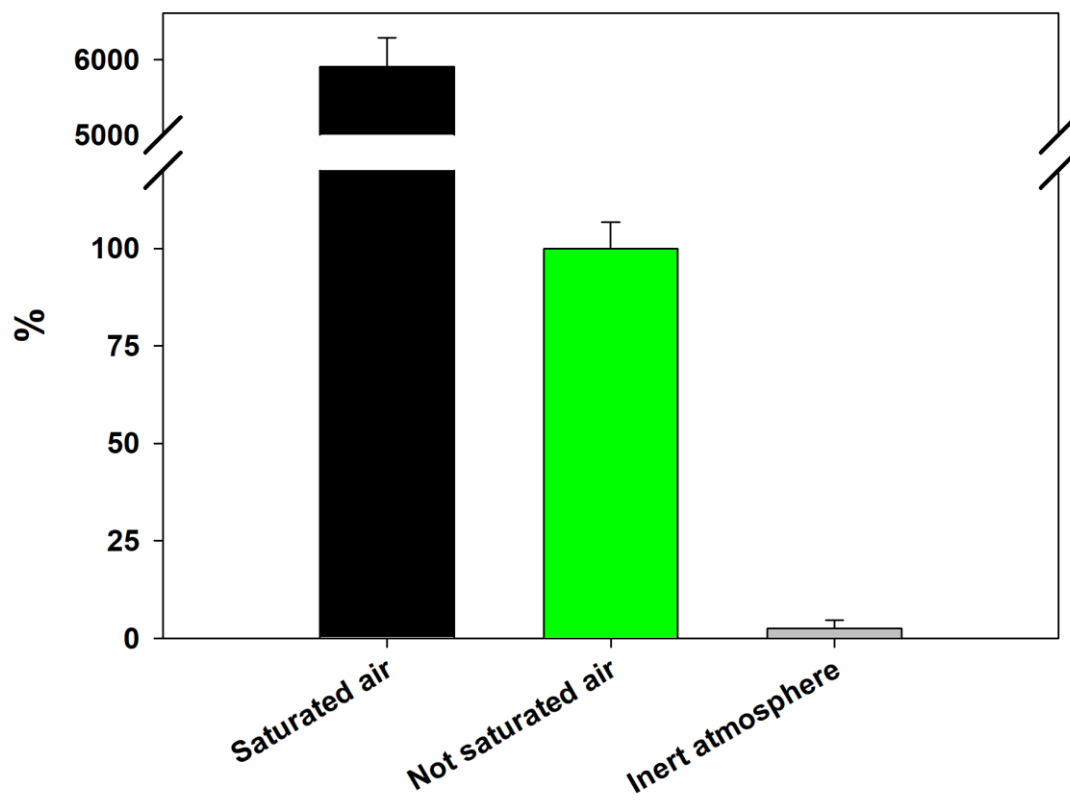

Supplement: FIG S3 [file mBio.00345-19-sf003.pdf]

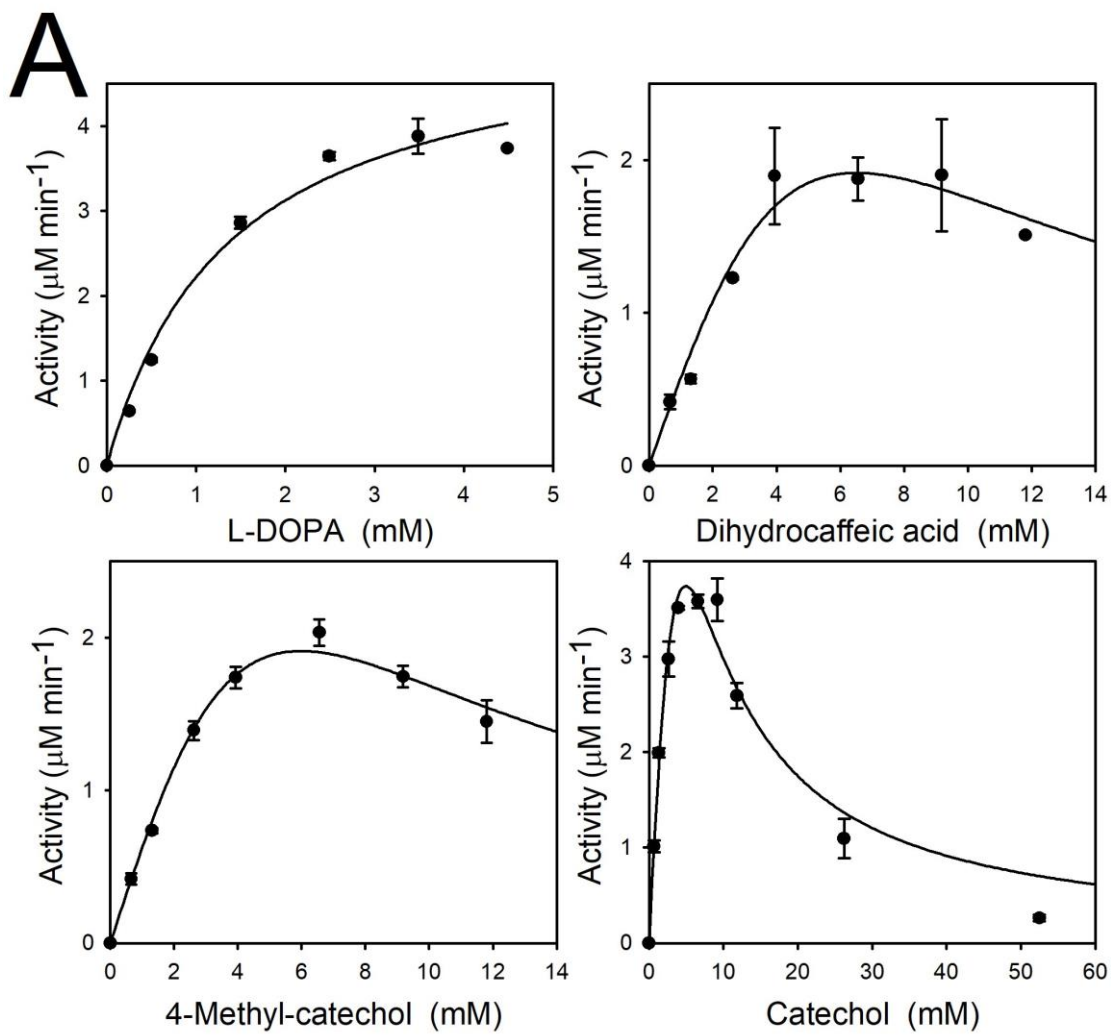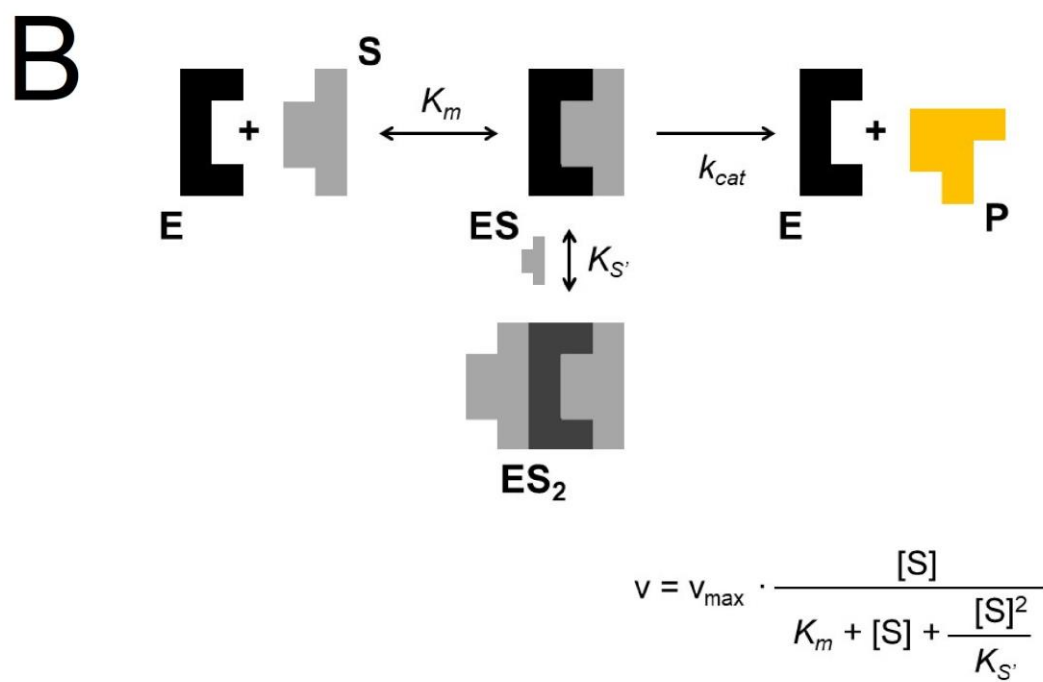

Supplement: FIG S4 [file mBio.00345-19-sf004.pdf]

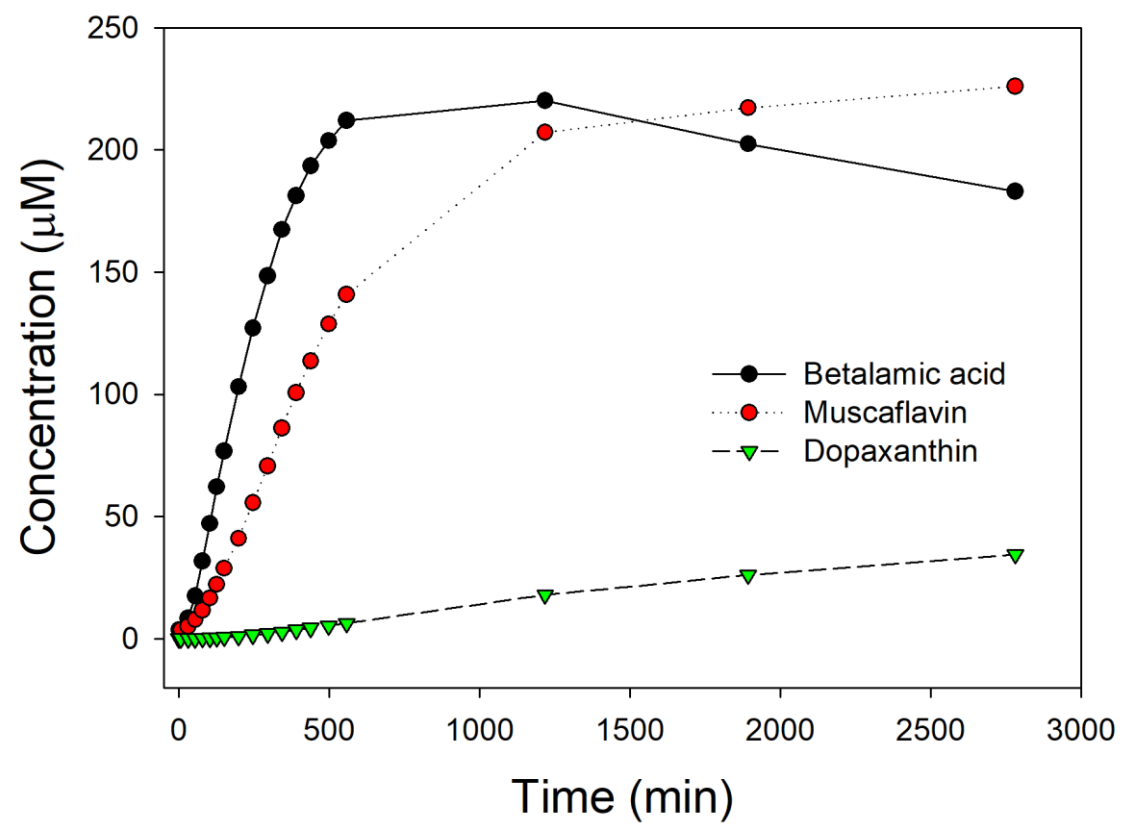

Supplement: FIG S6 [file mBio.00345-19-sf006.pdf]
