# Supplementary material for: First Betalain-Producing Bacteria Break the Exclusive Presence of the Pigments in the Plant Kingdom
Source: mBio. 2019 Mar 19;10(2):e00345-19. doi: 10.1128/mBio.00345-19 (PMC6426604; doi:10.1128/mBio.00345-19)
Supplement: TEXT S1 [file mBio.00345-19-s0001.pdf]

# SUPPLEMENTARY MATERIALS AND METHODS

## Structure modelling details

Structure homology were computed by the SWISS-MODEL (Swiss Institute of Bioinformatics, Biozentrum, University of Basel, Switzerland) homology server (1, 2) which relies on ProMod3, an inhouse comparative modelling engine based on OpenStructure (3). The SWISS-MODEL template library (SMTL version 2018-02-28, PDB release 2018-02-23) was searched with BLAST (4) and HHBlits (5) for evolutionary related structures matching the target sequence. PDB entry 2NYH was the best scored template for both alternative search algorithms.

ProMod3 extracts initial structural information from the selected template structure. Insertions and deletions, as defined by the sequence alignment, are resolved by first searching for viable candidates in a structural database. Final candidates are then selected using statistical potentials of mean force scoring methods. If no candidates can be found, a conformational space search is performed using Monte Carlo techniques. Non-conserved side chains are modelled using the 2010 backbone-dependent rotamer library from the Dunbrack group (6). The optimal configuration of rotamers is estimated using the graph-based TreePack algorithm (7) by minimising the SCWRL4 energy function (8). As a final step, small structural distortions, unfavourable interactions or clashes introduced during the modelling process are resolved by energy minimisation. ProMod3 uses the OpenMM library (9) to perform the computations and the CHARMM27 force field (10) for parameterisation.

In the present case a unique template (PDB entry 2NYH) was selected according to the quality of the resulting models. No other template represented alternative conformational states or covered different regions of the target protein. The process was

subjected to continuous evaluation within the Continuous Automated Model Evaluation (CAMEO) platform (11).

## Oligomeric modelling

The generated model was constructed as a dimer. For oligomeric modelling, in SWISS-MODEL, the quaternary structure annotation of the template is used to model the target sequence in its oligomeric form. The method used is based on a supervised machine learning algorithm, Support Vector Machines (SVM), which combines interface conservation, structural clustering, and other template features to provide a quaternary structure quality estimate (QSQE) (12). The QSQE score obtained was 0.49. QSQE is only computed if it is possible to build an oligomer and only for the top ranked templates, as in the present case. In this model the *in silico* results were corroborated by molecular characterization of the expressed and purified protein, experimentally determined to be a homodimer (please refer to main text for details).

**Model evaluation**

The GMQE and QMEAN parameter were determined to ascertain enough model quality. Results provide a satisfactory evaluation. The GMQE (Global Model Quality Estimation) was calculated as 0.62. This parameter provides a quality estimation which combines properties from the target-template alignment and the template search method. The resulting GMQE score is expressed as a number between 0 and 1, reflecting the expected accuracy of a model built with that alignment and template and the coverage of the target. Higher numbers indicate higher reliability.

The QMEAN parameter was calculated as -3.17. This is a composite estimator based on different geometrical properties and provides both global (entire structure) and local (per residue) absolute quality estimates on the basis of the single model used (13). The QMEAN score provides an estimate of the "degree of nativeness" of the structural features observed in the model on a global scale. It indicates whether the model is comparable to what one would expect from experimental structures of similar size. If QMEAN scores between zero and -4.0 indicate good agreement between the model structure and experimental structures of similar size (higher quality around 0.0). Scores of -4.0 or below are indicative of models of low quality. The obtained model is of enough quality to pass the standard evaluation parameters. The individual Z-scores comparing the interaction potential between Cβ atoms only, all atoms, the solvation potential and the torsion angle potential were similar to what one would expect from experimental structures of similar size, and also indicated the reliability of the model obtained.

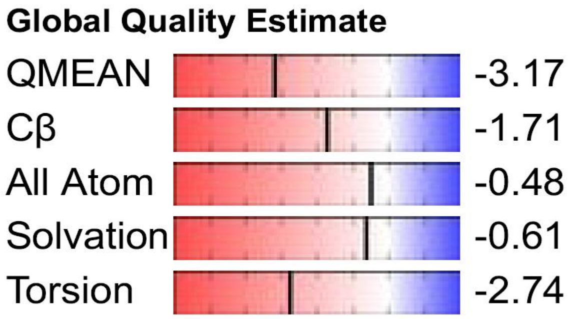


## REFERENCES

1. A. Waterhouse, M. Bertoni, S. Bienert, G. Studer, G. Tauriello, R. Gumienny, F.T. Heer, T.A.P. De Beer, C. Rempfer, L. Bordoli, R. Lepore, T. Schwede,. Nucleic Acids Res. 46:W296–W303, 2018, doi:10.1093/nar/gky427.
2. S. Bienert, A. Waterhouse, T.A.P. De Beer, G. Tauriello, G. Studer, L. Bordoli, T. Schwede. Nucleic Acids Res. 45:D313–D319, 2017. doi:10.1093/nar/gkw1132.
3. M. Biasini, T. Schmidt, S. Bienert, V. Mariani, G. Studer, J. Haas, N. Johner, A.D. Schenk, A. Philippsen, T. Schwede. Acta Crystallogr. Sect. D Biol. Crystallogr. 69:701–709, 2013. doi:10.1107/S0907444913007051.
4. C. Camacho, G. Coulouris, V. Avagyan, N. Ma, J. Papadopoulos, K. Bealer, T.L. Madden. BMC Bioinformatics. 10:421, 2009. doi:10.1186/1471-2105-10-421.
5. M. Remmert, A. Biegert, A. Hauser, J. Söding. Nat. Methods. 9:173–175, 2012. doi:10.1038/nmeth.1818.
6. M. V Shapovalov, R.L. Dunbrack. Structure. 19:844–858, 2011. doi:10.1016/j.str.2011.03.019.
7. C.B. McPake, C.B. Murray, G. Sandford. Tetrahedron Lett. 50:1674–1676, 2009. doi:10.1016/j.tetlet.2008.12.073.

8. G.G. Krivov, M. V. Shapovalov, R.L. Dunbrack. Proteins Struct. Funct. Bioinforma. 77:778–795, 2009. doi:10.1002/prot.22488.

9. P. Eastman, J. Swails, J.D. Chodera, R.T. McGibbon, Y. Zhao, K.A. Beauchamp, L.P. Wang, A.C. Simmonett, M.P. Harrigan, C.D. Stern, R.P. Wiewiora, B.R. Brooks, V.S. Pande. PLoS Comput. Biol. 13, 2007. doi:10.1371/journal.pcbi.1005659.

10. A.D. Mackerell, M. Feig, C.L. Brooks. J. Comput. Chem. 25:1400–1415, 2004. doi:10.1002/jcc.20065.

11. J. Haas, A. Barbato, D. Behringer, G. Studer, S. Roth, M. Bertoni, K. Mostaguir, R. Gumienny, T. Schwede. Proteins Struct. Funct. Bioinforma. 86:387–398, 2018. doi:10.1002/prot.25431.

12. M. Bertoni, F. Kiefer, M. Biasini, L. Bordoli, T. Schwede. Sci. Rep. 7, 2017. doi:10.1038/s41598-017-09654-8.

13. P. Benkert, M. Biasini, T. Schwede, Bioinformatics. 27:343–350, 2011. doi:10.1093/bioinformatics/btq662.
