# Supplementary material for: First Betalain-Producing Bacteria Break the Exclusive Presence of the Pigments in the Plant Kingdom
Source: mBio. 2019 Mar 19;10(2):e00345-19. doi: 10.1128/mBio.00345-19 (PMC6426604; doi:10.1128/mBio.00345-19)
Supplement: FIG S2 [file mBio.00345-19-sf002.pdf]

```
DODGd/1-142      MTPVPEPIRQIGTIGSYHAHVYFDGPDGRAAAEHLRAAIADRFAVRLGRWHEVPIGPHTL
fragment_1/1-30  MTPVPEPIRQIGTIGSYHAHVYFDGPDGRA------------------------------
fragment_2/1-22  --------RQIGTIGSYHAHVYFDGPDGRA------------------------------
fragment_3/1-17  ------------------------------------------------------------
fragment_4/1-11  ------------------------------------------------------------
fragment_5/1-10  MTPVPEPIRQ--------------------------------------------------
fragment_6/1-8   -----------------------------------RAAIADRF-----------------
fragment_7/1-6   ------------------------------------------------------------

DODGd/1-142      PMYQIAFDTALFATLVPWLMLNHQDLSILIHPNTRFPRRDHLRDGIWLGQPRALLGSRLP
fragment_1/1-30  ------------------------------------------------------------
fragment_2/1-22  ------------------------------------------------------------
fragment_3/1-17  ------------------------------------------RDGIWLGQPRALLGSRL-
fragment_4/1-11  ------------------------------------------RDGIWLGQPRA-------
fragment_5/1-10  ------------------------------------------------------------
fragment_6/1-8   ------------------------------------------------------------
fragment_7/1-6   --------------------------------------RDHLRD----------------

DODGd/1-142      EDAAEADGAGTPDTTPGGATPI
fragment_1/1-30  ----------------------
fragment_2/1-22  ----------------------
fragment_3/1-17  ----------------------
fragment_4/1-11  ----------------------
fragment_5/1-10  ----------------------
fragment_6/1-8   ----------------------
fragment_7/1-6   ----------------------
```
