# Supplementary material for: First Betalain-Producing Bacteria Break the Exclusive Presence of the Pigments in the Plant Kingdom
Source: mBio. 2019 Mar 19;10(2):e00345-19. doi: 10.1128/mBio.00345-19 (PMC6426604; doi:10.1128/mBio.00345-19)
Supplement: TABLE S1 [file mBio.00345-19-st001.pdf]

Table S1. Kinetic analysis of GdDODA with different substrates. Strong inhibition by excess of substrate was shown for dihydrocaffeic acid, 4-methyl-catechol and catechol.

Compound	Structure	V_{max} (µM·min^{-1})	K_m (mM)	Inhibition constant
L-DOPA	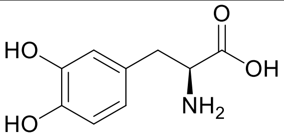	5.26	1.36	No inhibition
Dihydrocaffeic acid	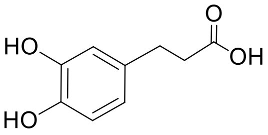	$63.60 \cdot 10^3$	$108.17 \cdot 10^3$	0.0004
4-Methyl-catechol	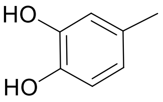	$70.99 \cdot 10^3$	$111.24 \cdot 10^3$	0.0003
Catechol	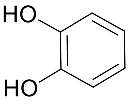	$32.60 \cdot 10^3$	$21.66 \cdot 10^3$	0.0011
