# Supplementary material for: First Betalain-Producing Bacteria Break the Exclusive Presence of the Pigments in the Plant Kingdom
Source: mBio. 2019 Mar 19;10(2):e00345-19. doi: 10.1128/mBio.00345-19 (PMC6426604; doi:10.1128/mBio.00345-19)
Supplement: FIG S5 [file mBio.00345-19-sf005.pdf]

Compound Mass Spectrum List Report - MS

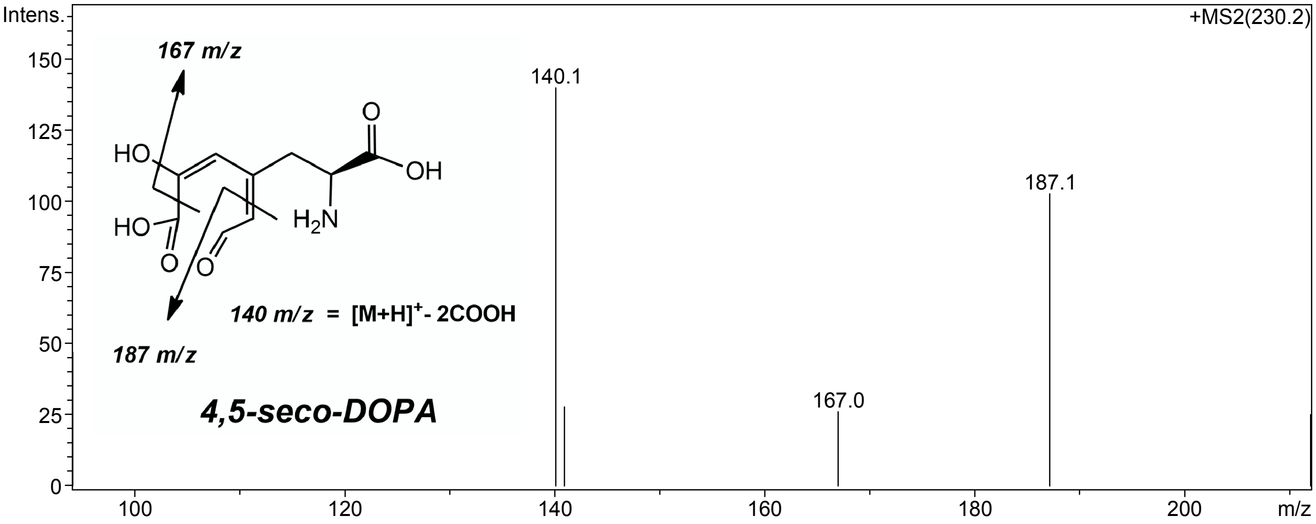

MS Peak List:

#	m/z	Res.	FWHM	I	I %	S/N
1	94.1			47	34	
2	140.1			140	100	
3	140.9			28	20	
4	167.0			26	19	
5	187.1			102	73	
6	211.9			25	18	

Compound Mass Spectrum List Report - MS

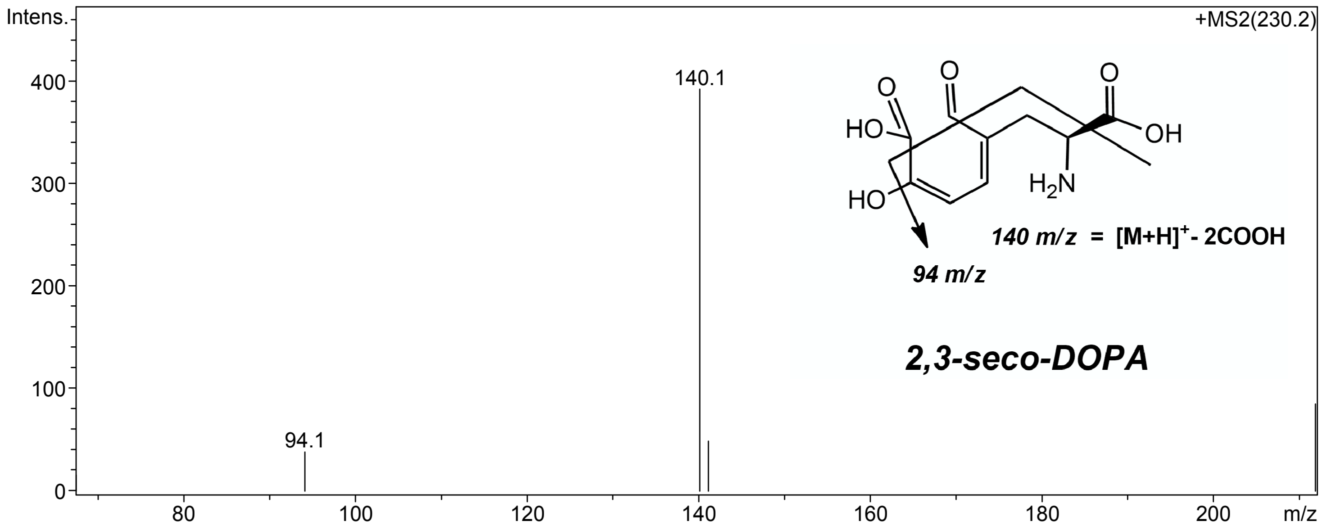

MS Peak List:

#	m/z	Res.	FWHM	I	I %	S/N
1	67.5			15	4	
2	94.1			37	10	
3	140.1			391	100	
4	141.2			48	12	
5	211.8			84	22	

Compound Mass Spectrum List Report - MS

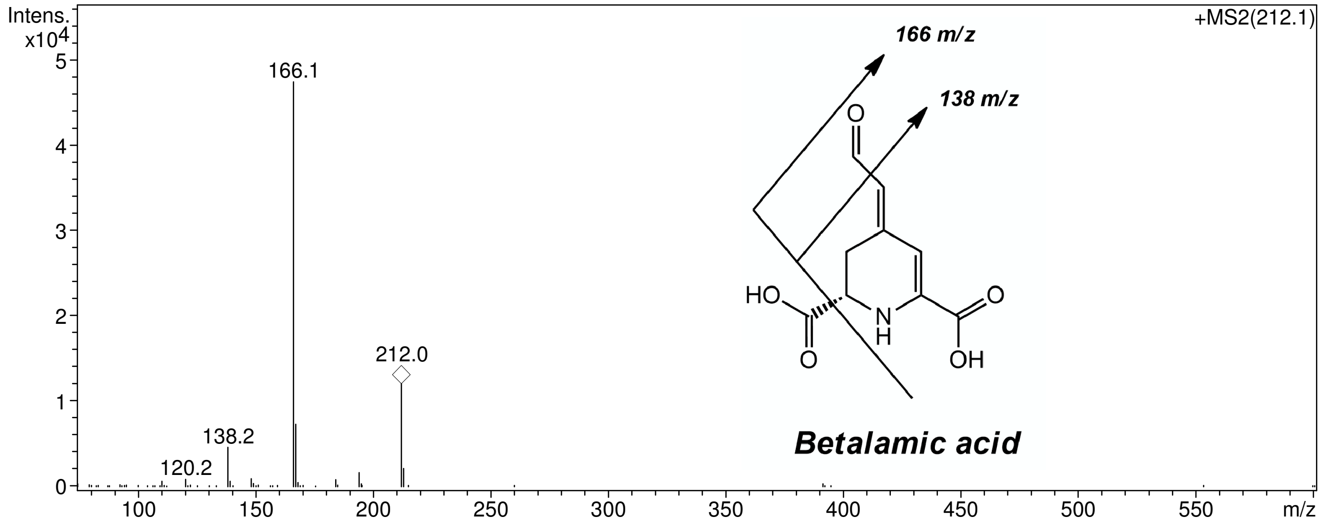

MS Peak List:

#	m/z	Res.	FWHM	I	I %	S/N
1	138.2			4518	10	
2	166.1			47362	100	
3	167.0			7204	15	
4	212.0			11977	25	

Compound Mass Spectrum List Report - MS

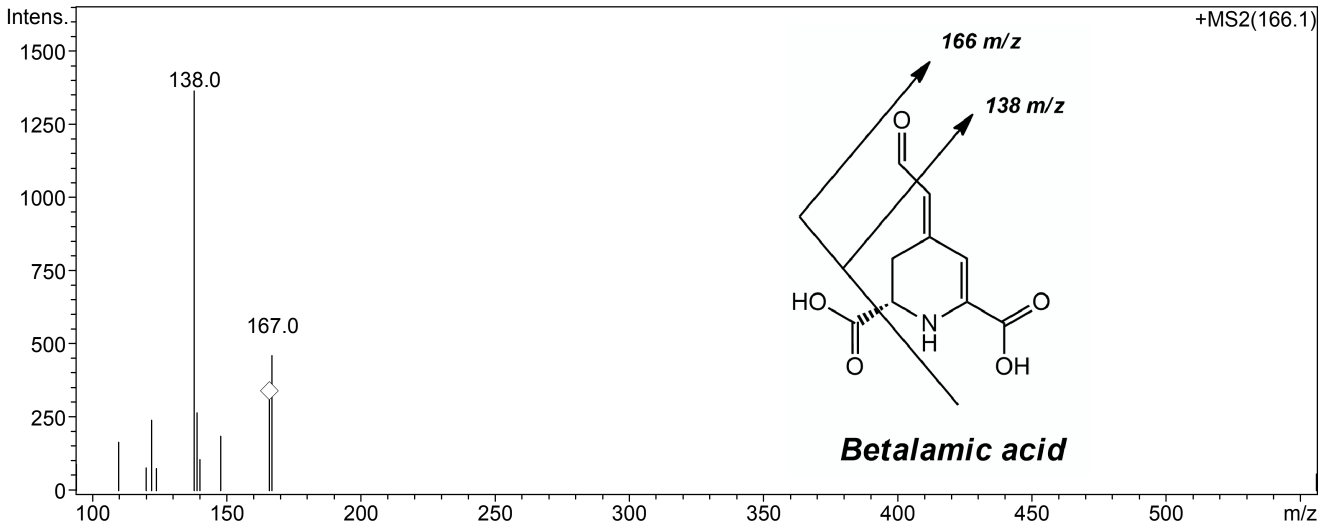

MS Peak List:

#	m/z	Res.	FWHM	I	I %	S/N
1	94.2			86	6	
2	110.0			163	12	
3	120.1			75	5	
4	122.1			237	17	
5	124.0			72	5	
6	138.0			1362	100	
7	139.0			263	19	
8	140.1			103	8	
9	148.0			184	14	
10	166.1			308	23	
11	167.0			458	34	
12	555.8			53	4	

Compound Mass Spectrum List Report - MS

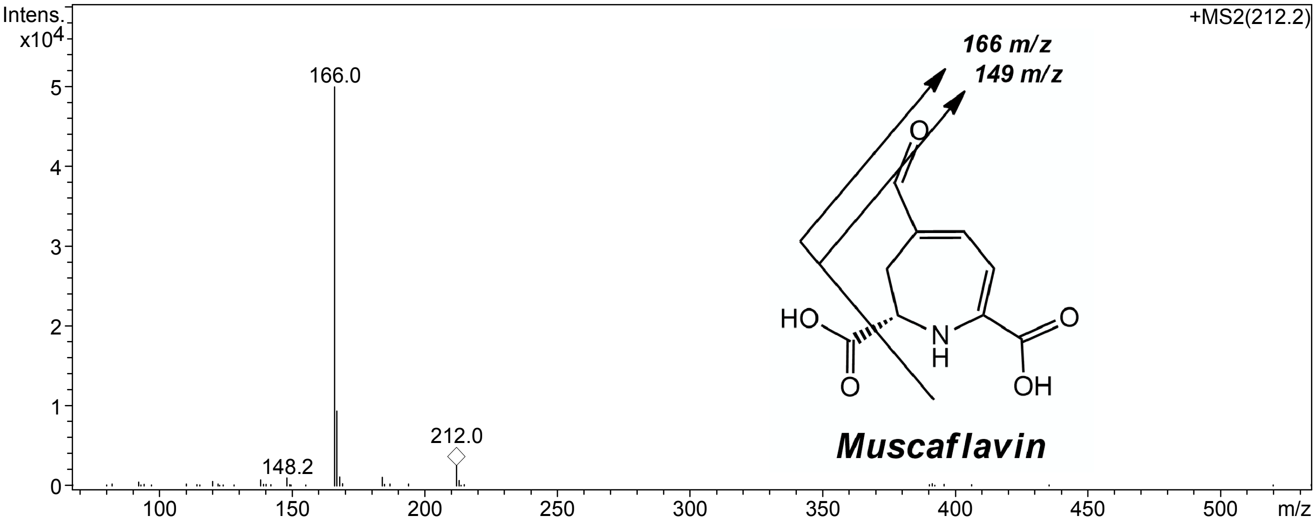

MS Peak List:

#	m/z	Res.	FWHM	I	I %	S/N
1	67.4			83	0	
2	80.3			61	0	
3	82.2			154	0	
4	92.2			419	1	
5	93.2			50	0	
6	94.3			128	0	
7	97.1			32	0	
8	110.3			176	0	
9	114.2			64	0	
10	115.4			22	0	
11	120.1			501	1	
12	122.2			154	0	
13	122.8			15	0	
14	124.1			38	0	
15	128.2			60	0	
16	138.2			687	1	
17	139.3			115	0	
18	140.2			111	0	
19	142.1			91	0	
20	148.2			933	2	
21	149.2			91	0	
22	149.7			53	0	
23	155.2			24	0	
24	166.0			49883	100	
25	167.0			9323	19	
26	168.0			1038	2	
27	169.0			221	0	
28	184.0			1011	2	
29	185.0			112	0	
30	187.0			165	0	
31	194.0			175	0	
32	212.0			2507	5	
33	213.0			617	1	
34	213.8			18	0	

Compound Mass Spectrum List Report - MS

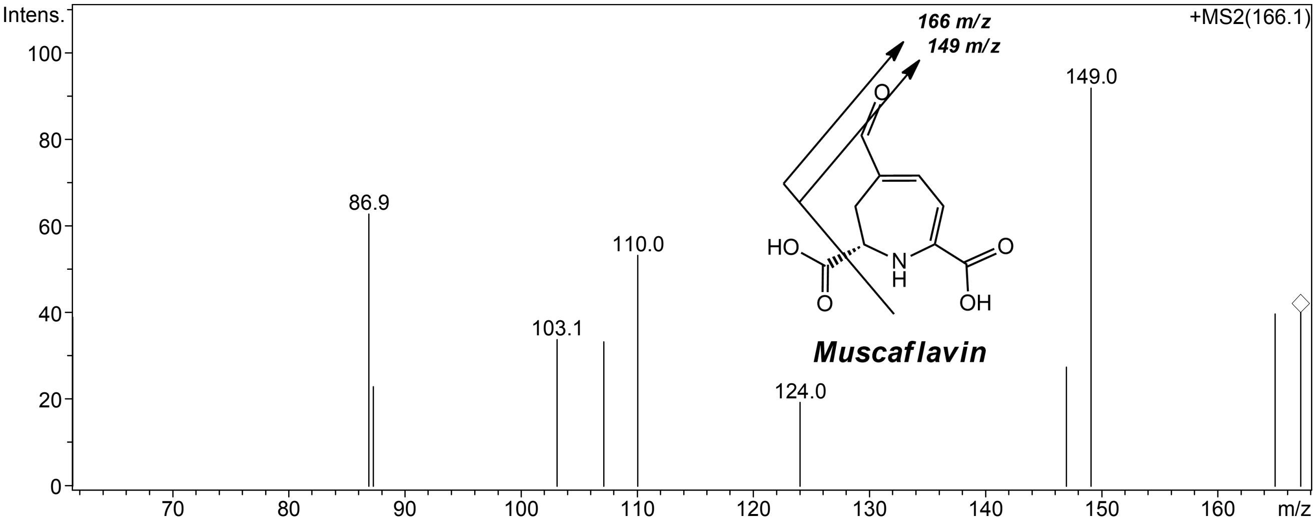

MS Peak List:

#	m/z	Res.	FWHM	I	I %	S/N
1	61.4			39	42	
2	86.9			63	68	
3	87.3			23	25	
4	103.1			34	37	
5	107.1			33	36	
6	110.0			53	58	
7	124.0			19	21	
8	146.9			27	30	
9	149.0			92	100	
10	164.9			40	43	
11	167.1			40	44	

Compound Mass Spectrum List Report - MS

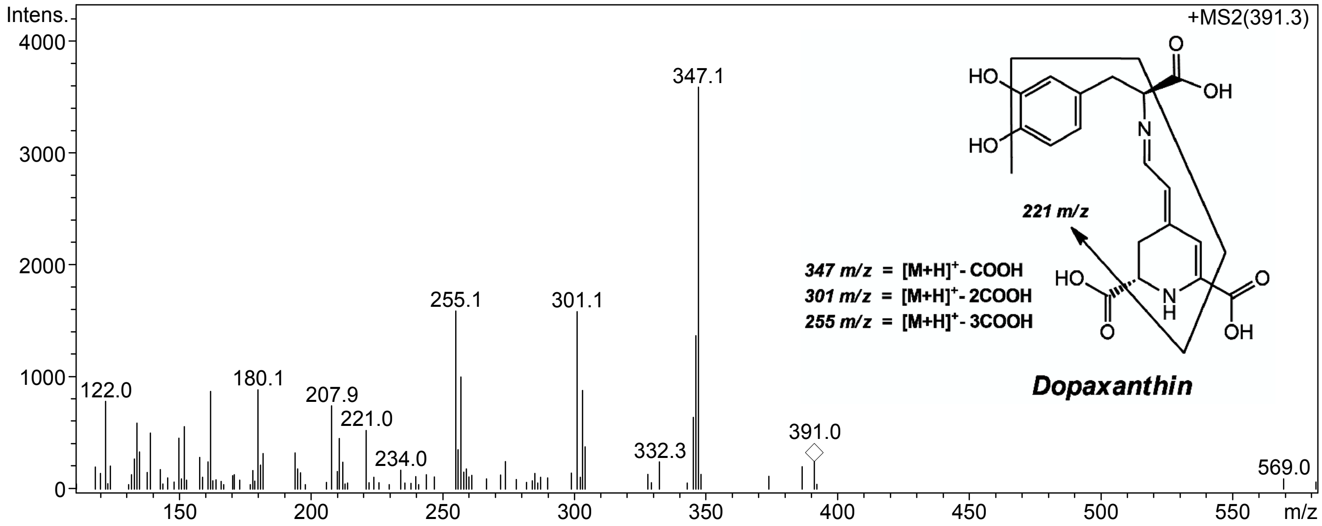

MS Peak List:

#	m/z	Res.	FWHM	I	I %	S/N
1	122.0			777	22	
2	134.0			582	16	
3	135.0			327	9	
4	139.1			495	14	
5	150.0			449	13	
6	152.1			553	15	
7	162.0			866	24	
8	180.1			880	25	
9	207.9			736	21	
10	210.8			445	12	
11	221.0			517	14	
12	255.1			1582	44	
13	256.0			346	10	
14	257.1			996	28	
15	301.1			1576	44	
16	303.2			876	24	
17	304.0			370	10	
18	345.1			633	18	
19	346.0			1364	38	
20	347.1			3582	100	
