# Supplementary material for: First Betalain-Producing Bacteria Break the Exclusive Presence of the Pigments in the Plant Kingdom
Source: mBio. 2019 Mar 19;10(2):e00345-19. doi: 10.1128/mBio.00345-19 (PMC6426604; doi:10.1128/mBio.00345-19)
Supplement: FIG S7 [file mBio.00345-19-sf007.pdf]

A

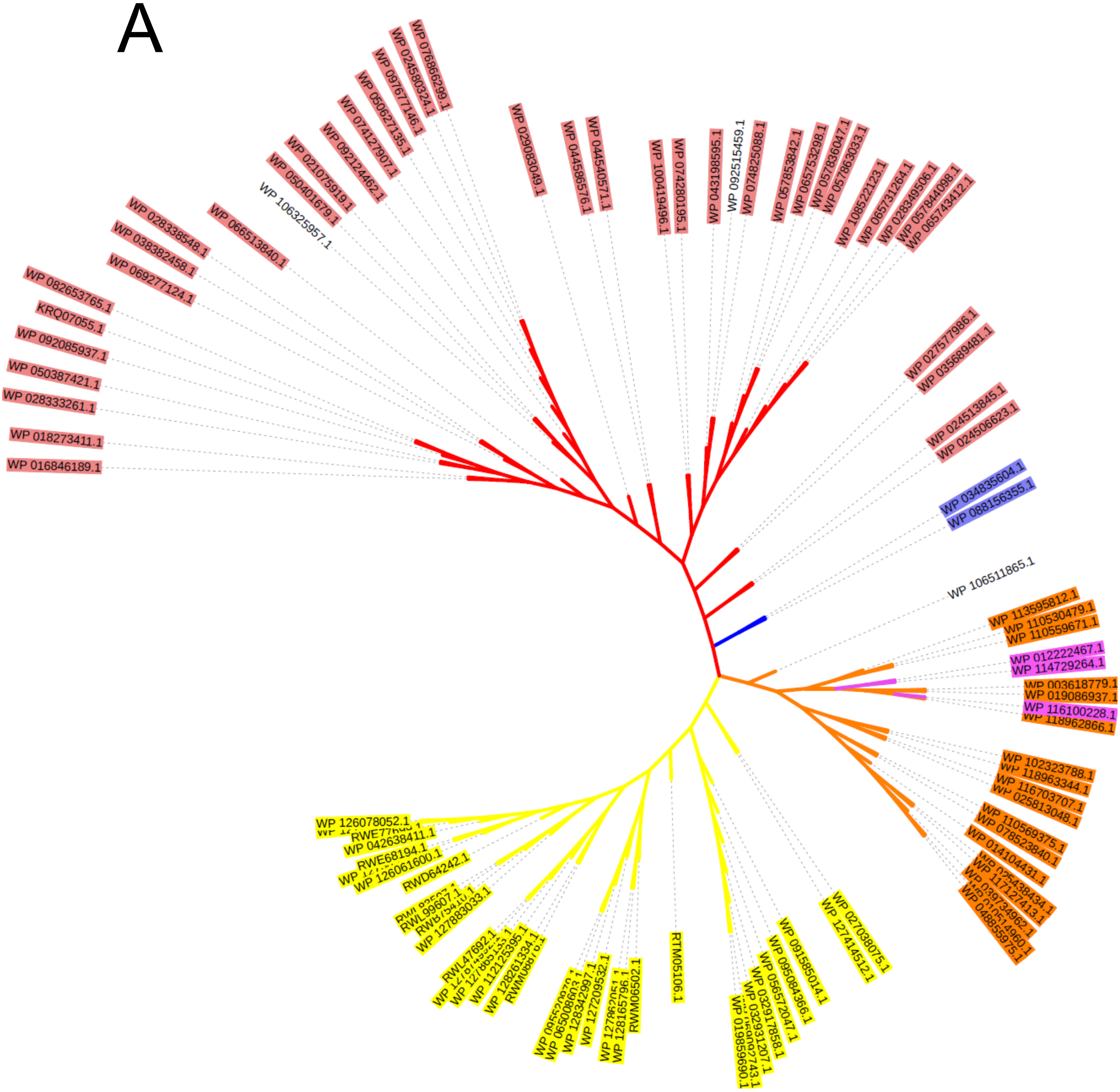
WP 076866299.1
WP 024580324.1
WP 097677146.1
WP 050627135.1
WP 074127907.1
WP 092124462.1
WP 021075919.1
WP 050401679.1
WP 106325957.1
WP 066513840.1
WP 028338548.1
WP 038382458.1
WP 069277124.1
WP 082653765.1
KRQ07055.1
WP 092085937.1
WP 050387421.1
WP 028333261.1
WP 018273411.1
WP 016846189.1
WP 029083049.1
WP 044596576.1
WP 044540571.1
WP 100419496.1
WP 074280195.1
WP 043198595.1
WP 092515459.1
WP 074825088.1
WP 057853842.1
WP 065753298.1
WP 057836047.1
WP 057863033.1
WP 108522123.1
WP 065731264.1
WP 028349506.1
WP 057844098.1
WP 065743412.1
WP 027577986.1
WP 035689481.1
WP 024513845.1
WP 024506623.1
WP 034835604.1
WP 088156355.1
WP 106511865.1
WP 113595812.1
WP 110530479.1
WP 110559671.1
WP 012222467.1
WP 114729264.1
WP 003618779.1
WP 019086937.1
WP 116100228.1
WP 118962866.1
WP 102323788.1
WP 118963344.1
WP 116703707.1
WP 025813048.1
WP 110569375.1
WP 078523840.1
WP 014104431.1
WP 025438434.1
WP 117127413.1
WP 039734962.1
WP 048855975.1
WP 126078052.1
WP 042638411.1
RWE68194.1
WP 126061600.1
RWD64242.1
RWL99607.1
WP 127883033.1
RWL47692.1
WP 112125395.1
WP 128261334.1
WP 128342997.1
WP 127209532.1
WP 128165796.1
RWM06502.1
RTM05106.1
WP 019856690.1
WP 032931207.1
WP 032917858.1
WP 065572047.1
WP 095084366.1
WP 091585014.1
WP 127414512.1
WP 027038075.1

B

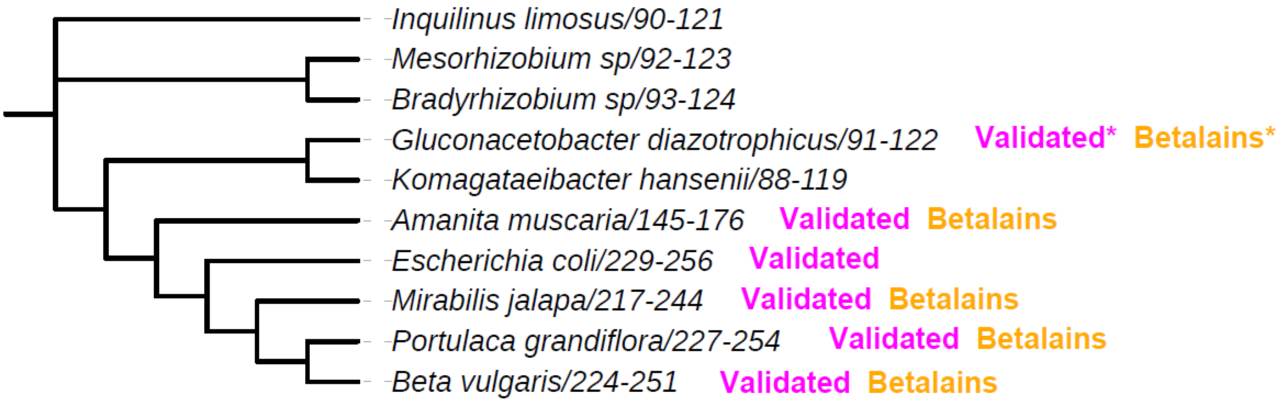

Validated: Enzyme functionally characterized on L-DOPA
Betalains: Betalamic acid derivatives found naturally

* *: Described in this work
